# Editorial: Impact of gut microbiota on neurogenesis and neurological diseases during early life

**DOI:** 10.3389/fnut.2025.1544128

**Published:** 2025-01-17

**Authors:** Tomás Cerdó, Cristina Campoy

**Affiliations:** ^1^Maimonides Biomedical Research Institute of Córdoba (IMIBIC), Reina Sofia University Hospital, University of Córdoba, Córdoba, Spain; ^2^Department of Pediatrics, School of Medicine, University of Granada, Granada, Spain; ^3^EURISTIKOS Excellence Centre for Pediatric Research, Biomedical Research Centre (CIBM), University of Granada, Granada, Spain; ^4^Federico Oloriz Neurosciences Institute, University of Granada, Granada, Spain; ^5^IBS-GRANADA, Instituto de Investigación Biosanitaria de Granada, Granada, Spain

**Keywords:** gut microbiota, neurodevelopment, early nutrition, microbiota gut-brain axis, neurogenesis

In this Research Topic several studies have analyzed the ability of nutritional microbial environment during perinatal and early life to reduce the risk of non-communicable and mental diseases later in life. Furthermore, the effect of the early gut microbiome development in the congenital alteration of brain functions has been studied, as well as the effect of prebiotic, probiotic, parabiotics and postbiotics supplementation on neurodevelopment, and neurodegenerative disorders; in addition, a study about the how neonatal infection influences on early brain development through the gut-brain axis is published within this Research Topic.

In the case of the gut microbiome and early environmental relationships with neurodevelopment, three studies have explored the link between early dysbiosis and long-term infant health. Beghetti et al., carried out a review delving into the relationship between both dynamic patterns and static features of the gut microbiota during preterm infants' early life and brain maturation, as well as neurodevelopmental outcomes in early childhood. Ozorio Dutra et al., explored associations between the infants' gut microbiome and early childhood behavior at 4 years of age in 19 children who were previously born with very low birth weight. They identified the bacterial taxa through a multivariate analysis by linear models, where *Veillonella dispar, Enterococcus, Escherichia coli*, and *Rumincococcus* were statistically significantly associated with later behavior at 4 years. Bezerra et al., wrote an opinion article which discussed how the double burden of malnutrition compounded with the environmental enteric dysfunction in growing children under adverse environments may negatively influence the intestinal microbiota homeostasis and hence the gastrointestinal tract-related melatonin function.

In the case of the association between gut microbiome and neurological disorders, Bao et al., characterized the gut microbial profiles in 32 children with Tourette syndrome (TS) and 29 healthy controls (HC), indicating a different gut microbial composition in children with TS with respect to HC, with multiple Gut-Brain Microbiota (GBM) neurotransmitter modules (Histamine degradation, Dopamine degradation, and 3,4-dihydroxyphenylacetic acid (DOPAC) synthesis) significantly increased. Moreover, combined physiotherapy (CES therapy and biofeedback training) was associated with a lower abundance of several genera and significant decreases in GBM neurotransmitter modules in patients following this treatment, indicating a possible improvement of clinical symptoms. Mendive Dubourdieu and Guerendiain, carried out a descriptive cross-sectional study analyzing the dietary intake and the gut composition of 30 children with autism spectrum disorder vs. 28 children with typical development, classified by their body mass index. Children with excess weight and ASD had lower *Roseburia* and *Faecalibacterium prausnitzii* and higher *Eubacterium ventricosum* and *Flavonifractor plautii* than the TD group with the same nutritional status. Moreover, they found positive and negative associations between the bacteria genus and species, and the nutrition in adjusted models, ASD/TD.

The effect of nutritional supplementation (prebiotic, probiotic, parabiotics and postbiotics) on early neurodevelopment was also explored by Rahim et al., by using 3,393 electronic databases with a total of 720 individuals between the ages of 2 and 17, as well as 112 adults ranging from 5 to 55 years old, all of whom had received a diagnosis of ASD. They observed that although there was no significant effect of such therapy on autism-related behavioral symptoms, psychobiotics had a significant effect on the brain connectivity through frontopolar power in beta and gamma bands mediated by chemicals and cytokines, such as TNF-α. In addition, Campbell et al., studied the influence of *in-utero* vitamins and minerals (BSM) exposure on infant temperament antenatally and for 12 months postpartum, in a cohort of 114 mother-infant dyads (45 infants exposed to BSM during pregnancy and 69 non-exposed). Results showed that BSM exposure did not significantly predict infant temperament, however, it may mitigate risks associated with antenatal depression. Furthermore, BSM-exposed infants displayed temperamental characteristics on par with typical pregnancies, supporting the safety of BSM treatment for antenatal depression.

Lastly, two studies in this Research Topic evaluated the function of the gut-brain axis in neurodegenerative disorders and neonatal infection. Vaia et al., carried out a mini-review that explored the intricate bidirectional relationship between gastrointestinal disorders and neurodegeneration in leukodystrophy infantile population, a disease relatively frequent in childhood causing neuro-motor disability, to affect the white matter of the brain. Tagi et al., performed a narrative review analyzing the state of the link between post-streptococcal autoimmune neuropsychiatric disorders (PANDAS) and gut microbiota composition in children. Notable changes included reduced microbial diversity and shifts in bacterial populations, which affect metabolic functions crucial for neuroinflammation. Moreover, elevated serum levels of sNOX2-dp and isoprostanes seem to indicate oxidative stress, while the presence of lipopolysaccharides (LPS) may contribute to neuroinflammation.

Overall, these findings might be important for developing gut microbiota-based therapeutic strategies for the treatment and/or prevention of behaviors or brain pathologies. These nine articles try to understand molecular mechanisms and pathways involved in microbiota-brain connections, elucidate some of the numerous sources of conflicting evidence and answer unanswered questions about the influence of intestinal dysbiosis on neurogenesis and neurological diseases during early life. However, it is important to emphasize that more studies are required to overcome the considerable gaps in transferring the results obtained in reductionist animal models to human clinical practice.

